# Knowledge, attitudes, and perceptions about antibiotic use and antimicrobial resistance among final year undergraduate medical and pharmacy students at three universities in East Africa

**DOI:** 10.1371/journal.pone.0251301

**Published:** 2021-05-07

**Authors:** Margaret Lubwama, Jackson Onyuka, Kirabo Tess Ayazika, Leoson Junior Ssetaba, Joseph Siboko, Obedi Daniel, Martha F. Mushi

**Affiliations:** 1 Department of Medical Microbiology, School of Biomedical Sciences, College of Health Sciences, Makerere University, Kampala, Uganda; 2 Department of Medical Laboratory Sciences, School of Health Sciences, Mount Kenya University, Thika, Kenya; 3 Department of Pharmacy, Bugando Medical Centre, Mwanza, Tanzania; 4 Department of Microbiology and Immunology, Catholic University of Health and Allied Sciences, Mwanza, Tanzania; National Institute for Infectious Diseases Lazzaro Spallanzani-IRCCS, ITALY

## Abstract

**Introduction:**

Proper measures to combat antimicrobial resistance development and spread in Sub Saharan Africa are very crucial bearing in mind the projected burden of antimicrobial resistance which is expected to be increase by 2050. Training of medical doctor and pharmacy students in antimicrobial stewardship is vital to combat antimicrobial resistance. This study was designed to evaluate the knowledge, attitude, and perception of final year medical and pharmacy students on antimicrobial use and antimicrobial resistance at three universities in Uganda, Kenya, and Tanzania.

**Methodology:**

A cross-sectional survey was carried out among final year undergraduate medical and pharmacy students at three universities in East Africa. A Self-administered questionnaire was developed which included dichotomous questions and questions using a 4-point Likert scale. The questions were based on knowledge and attitude about antibiotics, and preparedness to use antibiotics in clinical scenarios. Data were analyzed using STATA version 16 following the objective of the study.

**Results:**

Three hundred and twenty-eight final year students participated in the survey from MUK 75, MKU 75 and CUHAS 178. Slightly majority of participants were male 192(58.5%) and their median age was 25 [23 – 27] years. In general, 36.6% (120/328) of students had good overall total knowledge. More students at MUK had good knowledge compared to MKU, and CUHAS (72% vs, 40% vs. 20.2%; p<0.001). The mean scores for overall good total knowledge, general knowledge about antibiotics, knowledge about antibiotic resistance, and knowledge about antibiotic use in clinical scenarios were 58% (CI: 57%– 60%), 95% (CI: 94%– 97%), 54% (CI: 52% - 56%), and 46% (CI:44% - 48%) respectively. More pharmacy students compared to medical students had a good attitude and perception on antibiotic use (79.6% vs. 68.4%; p = 0.026). The students at CUHAS perceived being more prepared to use antibiotic in district hospitals compared to MKU and MUK (75.3% vs. 62.7% vs. 65.3%; p = 0.079). While two hundred and seventy (82.3%) students perceived knowing when to start antimicrobial therapy, 112 (34.2%) did not know how to select the appropriate antibiotic (p<0.0001), 97 (29.6%) did not know the antibiotic dose to give (p<0.0001), and 111 (33.8%) did not know when to switch form an intravenous antibiotic to oral regimen (p<0.0001).

**Conclusion:**

Final year students have low scores in knowledge about antimicrobial resistance and antibiotic use in clinical scenarios. This has exposed gaps in practical training of students, while they may feel confident, are not fully prepared to prescribe antibiotics in a hospital setting. A multidisciplinary and practical approach involving medical schools across the East African region should be undertaken to train final year undergraduate students in antimicrobial resistance and antimicrobial stewardship programs. Antimicrobial resistance and antimicrobial stewardship courses should be introduced into the curriculum of final year medicine and pharmacy programs.

## Introduction

Antimicrobial resistance is an evolving public health concern associated with increased morbidity and mortality [1]. Until 2050, it is estimated that antimicrobial resistance will have negative impacts on the global economy, mainly in low- and middle-income countries (LMIC), unless measures are taken to contain its threat [2, 3]. One of the drivers for antimicrobial resistance is the indiscriminate and irrational use of antibiotics driven by lack of knowledge and poor attitude of the prescribers and dispensers [2, 4]. Furthermore, populations of low-income countries are at a greater risk because of the ability to purchase antibiotics over the counter without prescription [5]. Therefore, the role of doctors and pharmacists in curbing antimicrobial resistance cannot be overemphasized. To enable optimization of infections’ treatment and reduce adverse events associated with antibiotic use during practice, it is prudent for doctors and pharmacists to receive ongoing training in antimicrobial stewardship beginning early enough in their education and career [4]. This training should include not only the fundamentals of diagnosis and management of infectious diseases, but also the association between antibiotic use and antimicrobial resistance [4].

Unlike in high-income countries where doctors and pharmacists who have specialized in infectious diseases are responsible for antibiotic prescriptions in sub-Saharan Africa (SSA), there are no such recognized training infectious diseases programs apart from South Africa [6], despite the current crisis of antimicrobial resistance [7]. Furthermore, due to limited human resources in health facility in low- and middle-income countries the final year undergraduate students begin to prescribe and/or dispense antibiotics with very minimal specialized supervision as soon as they start internship in health centers. Unfortunately, they may have neither an in-depth understanding of infectious diseases nor adequate knowledge on antibiotic use. In both developed and developing countries, studies have shown poor antibiotic knowledge among medical and pharmacy students, and that there is urgent need for effective training programs in the medical schools [8–12]. Indeed, potential causes of antibiotic abuse have been associated with a medical curriculum that has inadequately addressed the topic [9, 13]. In Africa, studies have shown that while students may have good knowledge on antibiotic use, they do not practice what they know [14]. Furthermore, it is noted that final year students are not confident prescribing antibiotics, and thus will benefit from antibiotic prescribing guidelines [15].

The aim of this study was to evaluate the knowledge, attitude, and perception of final year medical and pharmacy students on antimicrobial use and antimicrobial resistance at three universities in Uganda, Kenya, and Tanzania. We further assessed how prepared they perceived themselves to be to use antibiotics appropriately, and we compared this with their knowledge and attitude scores. In order to curb antimicrobial resistance, it is important to prepare the final year medical and pharmacy students to handle the task of appropriately using antibiotics. Therefore, it is of importance to understand their knowledge, attitude and perceptions about antibiotics. This situation will lead to robust antimicrobial stewardship programs in different health facilities in which these students will practice as junior doctors and pharmacist within the region.

## Methods

### Study design and setting

A cross-sectional survey was carried out among final year undergraduate medical and pharmacy students at three universities in East Africa to evaluate the knowledge, attitude, and perception of final year medical and pharmacy students on antimicrobial use and antimicrobial resistance. At Makerere University (MUK) in Kampala (Uganda) and Mount Kenya University (MKU) in Thika (Kenya), the study was carried out in October and November 2018. Furthermore, at Catholic University of Health and Allied Sciences (CUHAS) in Bugando (Tanzania) the study was carried out from May to July 2019. MUK College of Health Sciences is the oldest medical training University unit established in East Africa since 1924. MKU is the first private chartered university to offer Bachelor of Medicine and Bachelor of surgery in Kenya. CUHAS is located within Bugando Medical Center premises in Mwanza on the Bugando hill in the lake zone of Tanzania. CUHAS is the religious based second largest private university offering medial health professional training. At the moment the university offer 3 course at diploma level, 6 course at first degree, 7 course at second degree and PhD. In all three universities, as part of training, medical and pharmacy students receive basic training in microbiology and pharmacology early in the course in their second and third years. After the 5^th^ year for medical and 4^th^ year for pharmacy students of training, they will serve as junior doctors and pharmacist in various health centers in East Africa where they will be responsible for treating patients.

### Questionnaire development

The self-administered questionnaire was developed after reviewing other similar studies [16, 17]. It included dichotomous questions (Yes/No), and questions using a 4-point Likert scale (4 = strongly, 3 = Agree, 2 = Disagree, and 1 = Strongly disagree). The questions using the 4-point Likert scale were dichotomized such that correct answers for knowledge, attitudes, perceptions, and preparedness were noted if a participant agreed or strongly agreed (scale 3–4) in a positive question or disagreed or strongly disagreed (scale 1–2) in a negative question [9, 17]. A section on socio-demographic characteristics included questions regarding age, gender, university, and course of studying. Assessment of knowledge included 20 questions. The questions were further categorized into knowledge about antibiotics, knowledge about resistance, and knowledge about antibiotic use in clinical scenarios. Each question on knowledge about antibiotics carried one mark if passed and no mark if failed. Each question on knowledge about resistance carried two marks if passed and no mark if failed. Three marks was given if knowledge of all three mechanisms of resistance were correct. If at least one was wrong, no mark was given. Questions on knowledge about antibiotic use in clinical scenarios were divided into four categories: Diagnosis (4 questions), Prescribing antibiotics (3 questions), Antibiotic dose (1 question), and Switching antibiotics (1 question). If all questions were correct in each of the categories, 3 marks were given. If at least one question was incorrect, no mark was given. The total score for knowledge was 26 marks. Assessment of attitude included 5 questions. Each question in assessment of attitude carried one mark. The total score for attitude was 5 marks. Assessment of preparedness to prescribe antibiotics and manage bacterial infections included 8 questions. Each question in assessment of preparedness carried one mark if prepared, and no mark if not prepared. The total score for preparedness was 8 marks. The scores in all categories were converted to percentages. A pass mark of 60% was considered. (S1 Appendix) A score of <60% was considered poor knowledge while ≥60% was considered good knowledge. A score of <60% was considered poor attitude ≥60% was considered good attitude. A score of <60% was considered poorly prepared while ≥60% was considered well prepared.

### Data collection procedure

The questionnaire was pretested among continuing medical and pharmacy students. The pre-test was reviewed for irregularities. The lead researchers in the three different universities introduced the study to the university administration and students. The questionnaires were distributed randomly to the final year medical and pharmacy students. The students were informed in order to avoid aids while answering their own.

### Statistical analysis

Descriptive data were reported as frequencies and percentages. Continuous variables were described as median (interquartile range) and mean ±standard deviations (SD). Categorical variables were tabulated. Student’s t-test and chi-square test were used to assess differences between groups. A p-value of ≤0.05 (two-tailed) was considered statistically significant. Biostatistics analysis was carried out using STATA software (STATA Corp, College Station, TX, USA).

### Ethical consideration

Ethical clearance was obtained from the ethical committees of the three universities. Makerere University Kampala (School of Biomedical Sciences-592), Mount Kenya University (Mount Kenya University/Ethics Research Committee/0885), and Catholic University of Health and Allied Sciences (952/2019). Written informed consent was obtained from participants before participation in the study. Importantly, participation was voluntary, and confidentiality was maintained in this study.

## Results

Three hundred and twenty-eight final year students participated in the survey. Seventy-five of the students were from MUK (22.9%), 75 were from MKU (22.9%), and 178 were from CUHAS (54.3%). One hundred and ninety-two of the respondents were male (58.5%). The median age was 25 years [23 years– 27 years]. More than half of the students were in the Medicine course 196(59.8%) and 132 (40.2%) of the students were in the Pharmacy course. The socio-demographic characteristics of participants are shown in Table 1.

**Table 1:** Socio-demographic characteristics of participants.

Variable	General(328)	MKU(75)	MUK(75)	CUHAS(178)
Median Age [IQR] years	25[23–27]	23[22–24]	24[23–26]	25.5[23–27]
	N (%)	N (%)	N (%)	N (%)
Sex				
Male	192(58.5)	43(57.3)	48(64.0)	101(56.7)
Female	136(41.5)	32(42.7)	27(36.0	77(43.3)
Course				
Medicine	196 (59.8)	31 (41.3)	42 (56.0)	123 (69.1)
Pharmacy	132 (40.2)	44 (58.7)	33 (44)	55 (30.9)

### Knowledge on antibiotic use

In general, 36.6% (120/328) final year students had good overall total knowledge. More students at MUK had good overall total knowledge compared to MKU, and CUHAS (72% vs, 40% vs. 20.2%; p<0.001). While majority (325, 99.1%) of the final year students had good general knowledge about antibiotics, only 147 (44.8%) had good knowledge about antimicrobial resistance (p<0.001), and only 56 (17.1%) had good knowledge about antibiotic use in clinical scenarios (p<0.001). In knowledge about antibiotic use in clinical scenario questions, 60 (18.3%), 153 (46.7%), and 72 (22.0%) final year students answered correctly the questions on diagnosis, prescribing, and switching antibiotics respectively.

**Fig 1:**
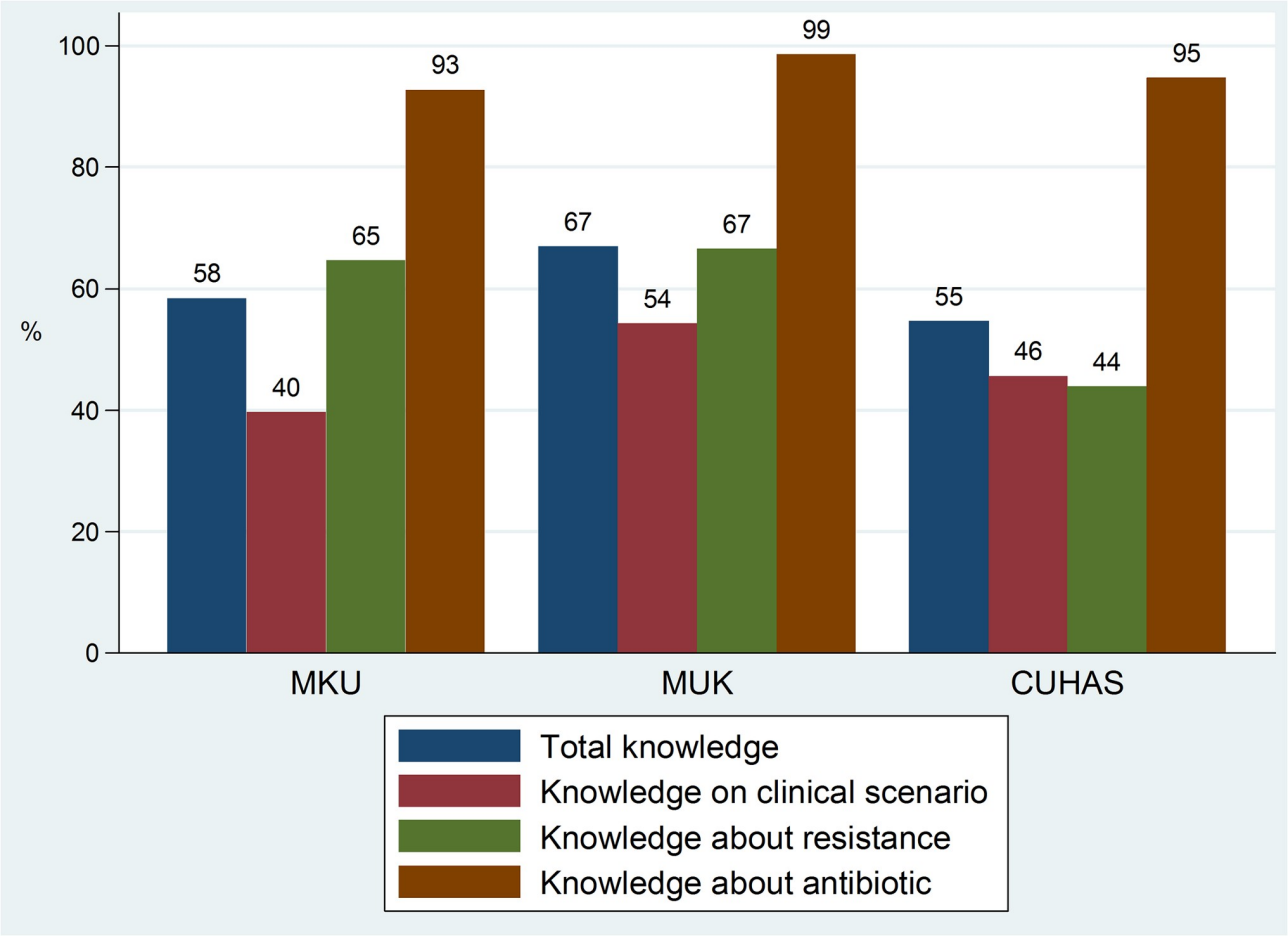
Mean scores of knowledge of final year students stratified by university.

The mean scores for overall good total knowledge, general knowledge about antibiotics, knowledge about antibiotic resistance, and knowledge about antibiotic use in clinical scenarios were 58% (CI: 57%– 60%), 95% (CI: 94%– 97%), 54% (CI: 52% - 56%), and 46% (CI:44% - 48%) respectively. The mean scores for overall good total knowledge, knowledge about resistance, and knowledge about antibiotic use in clinical scenarios were higher for students at MUK compared to the other universities (Fig 1). Mean score for overall total knowledge was below 60% pass mark in MKU (58%) and CUHAS (55%). The mean score for knowledge about resistance was below 60% in CUHAS (44%). In all three universities, in knowledge of antibiotic use in clinical scenarios, the mean score was below the 60% pass mark (Fig 1). The mean scores for pharmacy final students were higher compared to medical final year students for overall total knowledge (60% vs. 57%; p<0.001), knowledge about resistance (58.8% vs. 50.6%; p<0.0001), and knowledge about antibiotic use in clinical scenarios (47.2% vs. 45.7%; p<0.0001). Medical final year students had a higher mean score for general knowledge about antibiotics compared to the pharmacy students (95.8% vs. 94.4%; p<0.0001).

### Knowledge about antibiotics

More than 90% of students had good knowledge about antibiotics. Significant large proportion of students from MUK had good knowledge on antibiotics (three out of five questions were scored right by 100% of students). Importantly, up to 10 (5.6%) students did not agree that amoxicillin is an antibiotic. Also, 17.3% (n = 13) and 6.7% (n = 12) in MKU and CUHAS, respectively wrongly disagreed that antibiotics can kill normal flora. Furthermore, 14.7% (n = 11), 5.3% (n = 4) and 5.1% (n = 9) students from MKU, MUK, and CUHAS, respectively wrongly disagreed that antibiotics cause allergic reactions (Table 2).

**Table 2:** General knowledge about antibiotics.

	General	MKU	MUK	CUHAS	p-value
	N (%)	N (%)	N (%)	N (%)	
**1. There are many classes of antibiotics (Agree)**	317 (96.7)	74 (98.7)	75 (100)	168 (94.4)	0.042
**2. Amoxicillin is an antibiotic (Agree)**	317 (96.7)	74 (98.7)	74 (98.7)	169 (94.9)	0.175
**3. Panadol and Aspirin are antibiotics (Disagree)**	320 (97.6)	74 (98.7)	75 (100)	171 (96.1)	0.14
**4. Antibiotics can kill normal flora in the body (Agree)**	303 (92.4)	62 (82.7)	75 (100)	166 (93.3)	≤0.001
**5. Antibiotics can cause allergic reactions (Agree)**	304 (92.7)	64 (85.3)	71 (94.7)	169 (94.9)	0.021

### Knowledge about antimicrobial resistance

With regard to antimicrobial resistance, a total of 320 (97.6%) had knowledge that inappropriate use of antibiotics can lead to resistance. However, up to 98 (29.9%) wrongly agreed that prescribing broad spectrum antibiotics is always better even if there are narrower spectrum antibiotics that are effective. This was mostly observed in the students from CUHAS (p≤0.001). Furthermore, significantly, out of 325 students only 72 (22.2%) correctly disagreed that the mechanism resistance to methicillin in *S*. *aureus* is by efflux pumps with significant difference between the universities (30(40.0%) vs 13(17.3%) in MKU vs 29(16.6%)) in CUHAS, P ≤0.001). Out of 325 students, 273 (84.5%) correctly identified the mechanism of resistance to beta-lactams in *K*. *pneumoniae*, and out of 323 students, 267 (82.7%) correctly identified the mechanism of resistance to vancomycin resistant *E*. *faecalis* (Table 3).

**Table 3:** Knowledge on antimicrobial resistance.

	General	MKU	MUK	CUHAS	p-value
	N (%)	N (%)	N (%)	N (%)	
**1. Inappropriate use of antibiotics causes antibiotic resistance (Agree)**	320 (97.6)	74 (98.7)	72 (96)	174 (97.7)	0.554
**2. Better use of antibiotics will not have an impact on antimicrobial resistance (Disagree)**	183 (56.3)	60 (81.1)	60 (82.2)	63 (35.4)	≤0.001
**3. Prescribing broad spectrum antibiotics is always better even if there are narrower spectrum antibiotics that are effective (Disagree)**	230 (70.1)	69 (92.0)	64 (85.3)	97 (54.5)	≤0.001
**4. The mechanism of resistance to beta-lactams in *K*. *pneumoniae* is mainly enzymatic (Agree)**	273 (84.5)	66 (88.0)	60 (80.0)	147 (85.0)	0.388
**5. The mechanism of resistance to methicillin resistant *S*. *aureus* is by efflux pumps (Disagree)**	72 (22.2)	13 (17.3)	30 (40.0)	29 (16.6)	≤0.001
**6. The mechanism of resistance to vancomycin resistant *E*. *faecalis* is alteration of binding sites (Agree)**	267 (82.7)	62 (82.7)	61 (82.4)	144 (82.8)	0.998

### Knowledge about antibiotic use in clinical scenarios

In clinical scenario question, out of 324 students who attempted the question, up to 294 (90.7%) of the students correctly diagnosed a bacterial infection, but only 219 out of 319 students (88.8%), 199 out of 310 students (64.2), and 181 out of 313 students (57.8%) did not diagnose the upper respiratory bacterial infection as viral infection, pneumonia, or allergic reaction, respectively. Up to 51 (15.5%) students wrongly agreed that they would prescribe antibiotics immediately to prevent pneumonia in a 3-year-old with runny nose, mild fever, but no chills and no cough. Only slightly over two-thirds of the students (221 out of 327 students) correctly disagreed to prescribing intravenous antibiotic therapy for an ambulatory patient with a urinary tract infection (UTI) caused by sensitive *E*. *coli*. Only 72 out of 324 (22.2%) students correctly agreed that the best way to manage a patient with extended spectrum beta lactamase-producing *E*. *coli* infection was to switch the antibiotic from ceftriaxone to meropenem. Compared to students from MKU and MUK, a high proportion of students from CUHAS suggested switching to meropenem in a patient with ESBL-producing infection (3% vs. 8% vs. 34.7%; p≤0.001) (Table 4).

**Table 4:** Knowledge about antibiotic use in clinical scenarios.

	General	MKU	MUK	CUHAS	P-value
	N (%)	N (%)	N (%)	N (%)	
**1. a 10-year-old girl reports with a history of sore throat for two days. She has an exudate on her tonsils, with associated cervical lymphadenopathy. She has a temperature of 37.5°C, a slightly increased heart rate, normal respiratory rate, and a normal BP. Do you think she has**					
**a. Bacterial pharyngitis? (Agree)**	294 (90.7)	66 (91.7)	70 (94.6)	158 (88.8)	0.331
**b. Viral upper respiratory infection? (Disagree)**	109 (34.2)	21 (30.4)	28 (37.8)	60 (34.1)	0.647
**c. Pneumonia? (Disagree)**	199 (64.2)	55 (88.7)	66 (91.7)	78 (44.3)	≤0.001
**d. Allergy? (Disagree)**	181 (57.8)	50 (78.1)	61 (84.7)	70 (39.6)	≤0.001
**2. The doctor prescribes antibiotics for 10 days. However, on day three, the girl feels much better. What should she do? (Complete the dose of antibiotics as prescribed by the doctor)**	322 (98.2)	75 (100)	75 (100)	172 (96.6)	0.076
**3. A 3-year-old with runny nose, mild fever, but no chills and no cough. You would start antibiotics immediately to prevent pneumonia (Disagree)**	277 (84.5)	66 (89.2)	73 (97.3)	138 (77.5)	≤0.001
**4. A patient with a single positive blood culture with S. epidermidis isolated should be started on vancomycin immediately (No)**	248 (75.6)	44 (58.7)	65 (86.7)	139 (78.1)	≤0.001
**5. A 30-year-old female is diagnosed with a urinary tract infection. Culture and sensitivity results show *E*. *coli* that is susceptible to almost all the antibiotics. She can eat food and is ambulatory. Based on the results, she should start on intravenous therapy immediately (No)**	221 (67.6)	56 (75.7)	67 (89.3)	98 (55.1)	≤0.001
**6. A patient in the surgical ward develops a fever post-operatively. ESBL producing *E*. *coli* is isolated from a blood culture and a pus swab. The patient is currently on ceftriaxone. What tis the best way to manage the best way to manage this patient? (Switch to meropenem)**	72 (22.2)	3 (4.0)	8 (11.0)	61 (34.7)	≤0.001

1a-1d (Diagnosis), 2 (Antibiotic Dose), 3,4,5 (Prescribing antibiotics), 6 (Switching antibiotics).

### Attitude and perception on antibiotic use

In general, 239 (72.9%) had good a good attitude and perception on antibiotic use. More students at MKU had a good attitude and perception compared to MUK and CUHAS (80% vs. 72% vs. 70.2%; p = 0.274). More pharmacy students compared to medical students had a good attitude and perception on antibiotic use (79.6% vs. 68.4%; p = 0.026). While majority of the students used antibiotics up to three times in the previous year 282 (86%), only 128 (39.0%) students claimed not to buy antibiotics over the counter without a prescription. This was seen mostly among students from MKU 47(62.7%) and CUHAS 71(39.9%) than students from MUK 10(13.3%), p = ≤0.001, (Table 5). Overall, 214 (65.6%) of students agreed that antibiotics were overused in the hospitals they rotate in.

**Table 5:** Attitude and perception on antibiotic use.

Question (Answer)	General	MKU	MUK	CUHAS	p-value
	N (%)	N (%)	N (%)	N (%)	
**How many times did you use antibiotics in the last year? (Up to 3 times)**	282 (86.0)	72 (96.0)	64 (85.3)	146 (82)	0.014
**Do you take antibiotics when you have a fever? (No)**	274 (83.5)	71 (94.7)	71 (94.7)	132 (74.2)	≤0.001
**Do you stop taking antibiotics when you feel better? (No)**	241 (73.5)	60 (80)	52 (69.3)	129 (72.5)	0.303
**Do you buy antibiotics over the counter without a prescription? (No)**	128 (39.0)	47 (62.7)	10 (13.3)	71 (39.9)	≤0.001
**Do you keep leftover antibiotics for future use? (No)**	232 (70.7)	55 (73.3)	42 (56.0)	135 (75.8)	0.006

Although 223 (68.2%) students reported knowing what antimicrobial resistance means, only 88 (26.8%) were aware of the meaning of antimicrobial stewardship (p = <0.0001). More pharmacy than medicine students claimed to know the meaning of antimicrobial resistance (79.6% vs. 61.1%) and antimicrobial stewardship (33.3% vs. 22.5%). Furthermore, more pharmacy students perceived that their degree course had discussed the concepts of antimicrobial resistance (91% vs 78.1%; p = 0.002) and antimicrobial stewardship (59.9% vs. 55.4%; p = 0.430) compared to medical students. Overall, 315 (97.2%) final year students would like antimicrobial resistance and antimicrobial stewardship concepts to be discussed more in the course in their final year of medical school.

### Preparedness to use antibiotics appropriately

In general, 230 (70.1%) final year students perceived themselves to be prepared to use antibiotics appropriately while working at the district hospital. The students at CUHAS perceived being more prepared compared to MKU and MUK (75.3% vs. 62.7% vs. 65.3%; p = 0.079). Interestingly, while the students had not performed well in the knowledge questions, they still felt prepared to use antibiotics appropriately (p = 0.002). Nonetheless, among the students with good knowledge, more (60%) felt prepared to use antibiotics approximately. Most of the students (70.3%) who had a good attitude also felt prepared to use antibiotics appropriately (p = 0.912). While 270 (82.3%) students perceived knowing when to start antimicrobial therapy, 112 (34.2%) did not know how to select the appropriate antibiotic (p<0.0001), 97 (29.6%) did not know the antibiotic dose to give (p<0.0001), and 111 (33.8%) did not know when to switch form an intravenous antibiotic to oral regimen (p<0.0001) (Table 6).

**Table 6:** Preparedness to use antibiotics appropriately.

Question	General	MKU	MUK	CUHAS	p-value
	N (%)	N (%)	N (%)	N (%)	
**Do you feel prepared to**:					
**Know whether to give an antibiotic or not?**	275 (84.1)	66 (88.0)	58 (77.3)	151 (85.3)	0.164
**Know when to start antimicrobial therapy?**	270 (82.3)	61 (81.3)	60 (80.0)	149 (83.7)	0.755
**Know how to select the best antibiotic?**	216 (65.9)	47 (62.7)	41 (54.7)	128 (71.9)	0.025
**Know the dosage of antibiotic to give?**	231 (70.4)	52 (69.3)	48 (64.0)	131 (73.6)	0.303
**Know when to switch from an intravenous antibiotic to oral regimen?**	217 (66.2)	46 (61.3)	50 (66.7)	121 (66.2)	0.591
**Know the correct and relevant specimen to collect for an infection?**	224 (68.3)	44 (58.7)	47 (62.7)	133 (74.7)	0.021
**Distinguish between normal flora and a true pathogen from a microbiology report?**	212 (64.6)	35 (46.7)	40 (53.3)	137 (77.0)	≤0.001
**Understand resistance mechanisms based on a microbiology report?**	198 (60.3)	41 (54.7)	34 (45.3)	123 (69.1)	0.001

## Discussion

Antimicrobial resistance is a global health concern largely caused by inappropriate use of antibiotics therefore knowledge of prescribers and dispensers on antibiotic use is critical. This is among the few studies in East Africa to assess the knowledge, attitude, and preparedness of final year students on appropriate use of antibiotics in three universities in Uganda, Kenya and Tanzania.

Slightly more than a third of the respondents had overall good knowledge. Less than half had good knowledge on antimicrobial resistance, and less than a quarter had good knowledge on the use of antibiotics in clinical scenarios. Indeed, not all students attempted all the questions within the different categories. Similarly, in a nationwide, cross-sectional, multicenter survey carried out in Italy among young doctors it was demonstrated that almost all the participants were knowledgeable about different bacteria species and resistance mechanisms. However, less than half of the participants correctly responded to the clinical quizzes [18]. In a study carried out in India, most of the medical students had good knowledge about antibiotics, with up to 98.1% reporting correctly that antibiotics are useful for bacterial infections. As demonstrated in our study, knowledge of antibiotic use for different resistance mechanisms was poor [19]. In a similar study carried out in Nigeria in 2018, 64.7% of the respondents had good knowledge of antimicrobial resistance, and only 56.0% had good practice toward antimicrobial use [14].

Furthermore, our studies showed that pharmacy students were more knowledgeable in antimicrobial resistance and use of antibiotics in clinical scenarios than medical students based on the mean scores. The difference in curriculum detail in pharmacy vs medicine can explain the findings. A study carried out by Keijsers, et al. [20], found that pharmacy students were indeed more knowledgeable in basic pharmacology compared to medical students. Notably, pharmacists are increasingly playing significant roles in fighting infectious diseases especially in this era of antimicrobial resistance where antimicrobial combinations become more and more complicated due to the evolving epidemiology of organisms [21]. Importantly, the low scores for the medical students poses a threat in controlling infectious diseases as medical doctors are the ones who first encounter patients and direct the management of infections in the patients based on the different clinical scenarios. In most settings in lower- and middle-income countries the clinical pharmacist are very few or almost not there. The general pharmacists do not frequently encounter patients and therefore depends on the medical doctors’ antibiotic prescriptions to dispense antibiotics. The low scores in knowledge of resistance and knowledge of antibiotic use in clinical scenarios reflects the gap that is present between theory and practical. The poor practical skills can eventually lead to inappropriate antibiotic prescription, one of the drivers for antimicrobial resistance [22]. Furthermore, the students’ unpreparedness to use microbiology as guide for management of clinical scenarios using antibiotics highlights the gaps in their understanding of microbiology and its function in antimicrobial stewardship. A study in India showed a gap in the understanding of the microbiology career, and therefore the students, though they had career aspirations, were not prepared for them [23].

Over two-thirds of the respondents had good attitude and perception on antibiotic use. The students perceived that antibiotics are overused in hospitals they rotate in and acknowledged that inappropriate use of antibiotic causes antibiotic resistance. By having this knowledge, it is hopeful that the next prescribers and dispensers, with adequate training, will have a positive approach to antimicrobial resistance and antimicrobial stewardship policies [24]. However, interestingly, but not surprisingly, majority of the students (61%) reported that they bought antibiotics over the counter without a prescription from a doctor. In Africa, one of the factors associated with antimicrobial resistance is the ability to buy antibiotics over the counter without prescription [5]. Of the three universities, more students from MKU reported that they did not purchase antibiotics over the counter without prescription. In a study carried out in Rwanda, 59.8% of the respondents reported taking antibiotics only when prescribed by the doctor and 51.1% bought antibiotics with prescriptions. National level policies on prescribing and dispensing drugs should be emphasized in low- and middle-income countries. Furthermore, pharmacies and dispensaries should be regulated [22].

Most of the students responded confidently when asked about prescribing, including starting patients on antibiotics, giving an antibiotic or not, and knowing the correct dose to give. However, less than two-thirds of the respondents were not so confident about knowing how to select the best antibiotic and how to switch from one regimen to another. It is important to note, that students had low scores in the knowledge about antibiotic use in clinical scenarios, specifically in questions on diagnosis, prescribing, and switching antibiotics. Overconfidence has been associated with overprescribing which further leads to inappropriate use of antibiotics [15, 16]. Furthermore, our studies showed that the students were not very confident in microbiology fields that influence the choice of antibiotic prescribed. They reported not being able to interpret a culture report based on the resistance mechanisms, distinguish between normal flora and true pathogen or know the relevant specimen to collect in any given infection. Misdiagnosis and eventual misuse of antibiotics are consequences of lack of adequate microbiological input [25].

Although more students reported knowing more about antimicrobial resistance than antimicrobial stewardship, overall, majority of the students preferred both antimicrobial resistance and antimicrobial stewardship programs to be included in the curriculum of undergraduate training. A multidisciplinary approach, including pharmacy and microbiology, to curb antimicrobial resistance cannot be overemphasized.

This study evaluated antibiotic use in clinical scenarios, thus critically understanding the practical skills that the students have by the time they complete their degree course. This is important as we think of ways of creating curriculums that are multidisciplinary and standardized, and which can be applied across East Africa. The sample size of the students included in this study was small in MKU and MUK. Nonetheless, we were able to show critical baseline data that can be applied to larger implementation studies.

## Conclusion

Final year students have low scores in knowledge of resistance and antibiotic use in clinical scenarios. This has exposed gaps in practical training. While the students may feel confident, they are not fully prepared to prescribe antibiotics in a hospital setting. A multidisciplinary and practical approach involving medical schools across the East African region should be undertaken to train final year undergraduate students in antimicrobial resistance and antimicrobial stewardship programs. Antimicrobial resistance and antimicrobial stewardship focused courses should be introduced into the curriculum of final year medicine and pharmacy programs.

## Supporting information

S1 Appendix Questionnaire (with scores). (DOCX)
