# Supplementary material for: Knowledge, attitudes, and perceptions about antibiotic use and antimicrobial resistance among final year undergraduate medical and pharmacy students at three universities in East Africa
Source: PLoS One. 2021 May 7;16(5):e0251301. doi: 10.1371/journal.pone.0251301 (PMC8104438; doi:10.1371/journal.pone.0251301)
Supplement: S1 Appendix — (DOCX) [file pone.0251301.s001.docx]

| Questionnaire | | | |
| --- | --- | --- | --- |
| Sociodemographic characteristics | | | |
| Name of University | | | |
| MUK  MKU  CUHAS | | | |
| Course | | | |
| Medicine  Pharmacy | | | |
| Age | | | |
| Gender | | | |
| Male  Female | | | |
| **Knowledge questions (20 questions)** | | | |
| **Knowledge about antibiotics** | | | |
| Question | Strongly Agree/Agree | Strongly disagree/disagree | Score |
| 1. There are many classes of antibiotics | correct | wrong | 1 |
| 2. Amoxicillin is an antibiotic | correct | wrong | 1 |
| 3. Panadol and Aspirin are antibiotics | wrong | correct | 1 |
| 4. Antibiotics can kill normal flora in the body | correct | wrong | 1 |
| 5. Antibiotics can cause allergic reactions | correct | wrong | 1 |
| **Total score for Knowledge about antibiotics** | | | **5 marks** |
| **Knowledge about resistance** | | | |
| 6. Inappropriate use of antibiotics causes antibiotic resistance | correct | wrong | 2 |
| 7. Better use of antibiotics will not have an impact on antimicrobial resistance | wrong | correct | 2 |
| 8. Prescribing broad spectrum antibiotics is always better even if there are narrower spectrum antibiotics that are effective | wrong | correct | 2 |
| 9. The mechanism of resistance to beta-lactams in *K. pneumoniae* is mainly enzymatic | correct | wrong | 3 (if all three are correct)  0 (if at least one is wrong) |
| 10. The mechanism of resistance to methicillin resistant *S. aureus* is by efflux pumps | wrong | correct |  |
| 11. The mechanism of resistance to vancomycin resistant *E. faecalis* is alteration of binding sites | correct | wrong |  |
| **Total score for Knowledge about resistance** | | | **9 marks** |
| **Knowledge about antibiotic use in clinical scenarios** | | | |
| **Diagnosis** | | | |
| 10yr old girl reports with a history of sore throat for two days. She has an exudate on her tonsils, with associated cervical lymphadenopathy. She has a temperature of 37.5˚C, a slightly increased heart rate, normal respiratory rate, and a normal BP. Do you think she has |  |  |  |
| 12. Bacterial pharyngitis? | correct | wrong | 3 (if all four are correct)  0 (if at least one is incorrect) |
| 13. Viral upper respiratory infection? | wrong | correct |  |
| 14. Pneumonia? | wrong | correct |  |
| 15. Allergy? | wrong | correct |  |
| **Antibiotic dose** | | | |
| 16. The doctor prescribes antibiotics for 10 days. However, on day three, the girl feels much better. What should she do? (Complete the dose of antibiotics as prescribed by the doctor) | correct | wrong | 3 |
| **Prescribing antibiotics** | | | |
| 17. A 3yr old with runny nose, mild fever, but no chills and no cough. You would start antibiotics immediately to prevent pneumonia | wrong | correct | 3 (if all are correct)  0 (if at least one is incorrect) |
| 18. A patient with a single positive blood culture with *S. epidermidis* isolated should be started on vancomycin immediately | wrong | correct |  |
| 19. A 30yr old female is diagnosed with a urinary tract infection. Culture and sensitivity results show *E. coli* that is susceptible to almost all the antibiotics. She can eat food and is ambulatory. Based on the results, she should start on intravenous therapy immediately | wrong | correct |  |
| **Switching antibiotics** | | | |
| 20. A patient in the surgical ward develops a fever post-operatively. ESBL producing E. coli is isolated from a blood culture and a pus swab. The patient is currently on ceftriaxone. What tis the best way to manage the best way to manage this patient? | | | |
| Continue with ceftriaxone | Wrong | Correct | 3 (if correctly answers switch to meropenem) |
| Continue with ceftriaxone and add gentamycin | Wrong | Correct |  |
| Stop ceftriaxone | Wrong | Correct |  |
| Switch to vancomycin | Wrong | Correct |  |
| Switch to meropenem | correct | Wrong |  |
| **Total score for Knowledge about antibiotic use in clinical scenarios** | | | **12 marks** |
| **Overall Total Score for Knowledge** | | | **26 marks** |
| **Attitude Questions (5 questions)** | | | |
| 1. How many times did you use antibiotics in the last year? |  |  |  |
| Up to 3 times | good attitude |  | 1 (if good attitude)  0 (if bad attitude) |
| 4-5 times | bad attitude |  |  |
| >5 times | bad attitude |  |  |
| 2. Do you take antibiotics when you have a fever? |  |  |  |
| No | Good attitude |  | 1 (if good attitude) |
| Yes | Bad attitude |  |  |
| 3. Do you stop taking antibiotics when you feel better? |  |  |  |
| No | Good attitude |  | 1 (if good attitude) |
| Yes | Bad attitude |  |  |
| 4. Do you buy antibiotics over the counter without a prescription? |  |  |  |
| No | Good attitude |  | 1 (if good attitude |
| Yes | Bad attitude |  |  |
| 5. Do you keep leftover antibiotics for future use? |  |  |  |
| No | Good attitude |  | 1 (if good attitude) |
| Yes | Bad attitude |  |  |
| **Total score for attitude** | | | **5 marks** |
| **Preparedness to prescribe antibiotics (8 questions)** | | | |
| Do you feel prepared to: | | | |
| 1. Know whether to give an antibiotic or not? | prepared | Not prepared | 1 (if prepared) |
| 2. Know when to start antimicrobial therapy? | prepared | Not prepared | 1 (if prepared) |
| 3. Know how to select the best antibiotic? | prepared | Not prepared | 1 (if prepared) |
| 4. Know the dosage of antibiotic to give? | prepared | Not prepared | 1 (if prepared) |
| 5. Know when to switch from an intravenous antibiotic to oral regimen? | prepared | Not prepared | 1 (if prepared) |
| 6. Know the correct and relevant specimen to collect for an infection? | prepared | Not prepared | 1 (if prepared) |
| 7. Distinguish between normal flora and a true pathogen from a microbiology report? | prepared | Not prepared | 1 (if prepared) |
| 8. Understand resistance mechanisms based on a microbiology report? | prepared | Not prepared | 1 (if prepared) |
| **Total score for preparedness** | | | **8 marks** |
